# Analysis of metabotropic glutamate receptor 7 as a potential substrate for SUMOylation

**DOI:** 10.1016/j.neulet.2011.01.032

**Published:** 2011-03-24

**Authors:** Kevin A. Wilkinson, Jeremy M. Henley

**Affiliations:** Medical Research Council Centre for Synaptic Plasticity, School of Biochemistry, Medical Sciences Building, University of Bristol, University Walk, Bristol BS8 1TD, UK

**Keywords:** SUMO, SUMOylation, mGluR7, Ubc9, Ubiquitin, Post-translational modification, GPCR, mGluR7, Metabotropic glutamate receptor

## Abstract

Group III metabotropic glutamate receptors (mGluRs) undergo post-translational modification by SUMO in *in vitro* assays but the SUMOylation of full-length mGluRs in mammalian cells has not been reported. Here we investigated SUMOylation of mGluR7 in HEK293 cells and primary cortical neurons in an attempt to confirm SUMOylation and define physiological effects on mGluR7 function. Using a recombinant bacterial expression assay we validated *in vitro* SUMOylation of the C-terminal domain of mGluR7 by both SUMO-1 and SUMO-2 and show that a single lysine residue (K889) in mGluR7 is required for SUMOylation. However, using a range of approaches, we were unable to detect SUMOylation of full-length mGluR7 in either heterologous cells or neurons. Further, we observed no differences in receptor stability or surface expression between wild-type and a non-SUMOylatable point mutant mGluR7. Thus, our results question whether mGluR7, and by implication other group III mGluRs, are physiologically relevant neuronal SUMO substrates.

## Research highlights

► mGluR7 is a presynaptic GPCR that modulates transmission. ► We confirm that SUMO can be conjugated to c-terminal fragments of mGluR7 *in vitro*. ► We show that mutation of lysine 889 abolishes SUMOylation in *in vitro* assays. ► We cannot detect any biochemical or functional evidence for SUMOylation of full-length mGluR7. ► Our data question if native mGluR7 is a physiologically relevant SUMO substrate.

Metabotropic glutamate receptors (mGluRs) are G protein-coupled receptors (GPCRs) that regulate synaptic function. Group III mGluRs (mGluR4, mGluR6, mGluR7 and mGluR8) are mostly presynaptic and act as autoreceptors to inhibit glutamate release via several pathways. With the exception of mGluR6, which is largely restricted to postsynaptic sites of retinal rod bipolar cells, group III mGluRs are expressed widely throughout the brain at both the RNA and the protein levels and, interestingly, they are present at both glutamatergic and GABAergic terminals (reviewed in Ref. [14]).

SUMO (Small Ubiquitin-like MOdifier) proteins are ∼11 kDa proteins that can be covalently conjugated to lysine residues in target proteins, altering the biochemical and/or functional properties of the modified protein. Three SUMO paralogues (SUMO-1–3) have been identified in vertebrate brain. SUMO-1 was first reported as a protein conjugated to the nuclear pore complex protein RanGAP [12] and several hundred targets of SUMOylation have since been identified. SUMO-2 and SUMO-3 differ by just three N-terminal amino acids but they share only ∼50% sequence identity to SUMO-1 [5]. Proteins are SUMOylated via an enzymatic cascade analogous to ubiquitination. Briefly, SUMO proteins are first activated by the action of an E1 ‘activating’ enzyme, which passes the activated SUMO to the E2 ‘conjugating’ enzyme. The only E2 enzyme in the SUMOylation pathway is Ubc9, which usually, but not always, in conjunction with an E3 ‘ligase’ enzyme, catalyses the SUMO conjugation to the substrate (reviewed in Ref. [21]).

Yeast two-hybrid assays using the intracellular C-terminus of the group III receptor mGluR8 isolated PIAS1 (Protein Inhibitor of Activated STAT) [20], an E3 component of the SUMOylation pathway. Those workers went on to show that PIAS1 interacted with each of the group III mGluRs *in vitro* and that the C-terminus of mGluR8 could be SUMOylated in HEK293 cells when expressed as a GST-fusion protein [20]. More recently, we demonstrated that each of the group III mGluRs was SUMOylated in a recombinant bacterial *in vitro* SUMOylation assay [23]. Subsequent yeast two-hybrid assays using mGluR8b as bait isolated Ubc9 as well as the SUMO E3 enzymes PIAS1 and PIAS3 as interacting proteins [19]. Interestingly, that study also identified Ubc12 as a potential mGluR8 interactor [19]. Ubc12 is a conjugating enzyme specific to another ubiquitin-like protein, Nedd8 (for review see [16]), suggesting that neddylation, in addition to SUMOylation and ubiquitination may play a role in the regulation of neurotransmitter receptors at the synapse.

Despite these data demonstrating SUMOylation of C-terminal domain constructs, SUMO modification of full-length group III mGluRs has not been reported and the functional consequences of this modification, should it occur, are unclear. We therefore sought to identify the site of modification in mGluR7, construct a full-length SUMOylation deficient mutant and analyse the consequences. We confirm robust SUMOylation of the C-terminus of mGluR7 *in vitro* and show that this is prevented by mutation of the lysine residue K889. However, we were unable to detect SUMO modification of the full-length receptor in either heterologous cells or neurons. Further, no differences in receptor expression levels, signalling or surface expression were detected between wild-type mGluR7 and the non-SUMOylatable mutant. Thus, our data question whether mGluR7, and by implication other group III mGluRs are genuine SUMO substrates in mammalian cells.

*Mutagenesis*: mGluR7(K889R) was made using Quikchange II XL site-directed mutagenesis (Stratagene) using either pRK5-SEP-mGluR7 (for mammalian expression) or pGEX-mGluR7-ct (for bacterial expression) as a template. The presence of the mutation was confirmed by DNA sequencing.

*Cell culture*: HEK293 cells and cortical neurons were cultured as described previously [2]. Briefly, cortical neurons were plated in Neurobasal media (Gibco), containing 5% horse serum, B27 supplement, glutamax and penicillin/streptomycin at a density of 0.5 million per 35 mm dish. After 24 h the media were changed and neurons were used at ∼14 DIV.

*mGluR7 solubilisation*: Cultured neurons or HEK293 cells were lysed in RIPA buffer (10 mM Tris–HCl pH 7.5, 150 mM NaCl, 1% Triton X-100, 1% sodium deoxycholate, 0.1% SDS, 20 mM NEM, containing complete mammalian protease inhibitors (Roche)), sonicated for 10 s and proteins solubilised by rotation on a wheel at 4 °C for 1 h. Lysates were then centrifuged at 16,000 × *g* for 20 min and the supernatant collected.

*SDS-PAGE and Western blotting*: Proteins were separated using 8–12% acrylamide gels and blotted onto Immobilon membranes for Western blotting with the antibodies indicated. Blots were quantified by densitometry of at least three separate experiments using NIH ImageJ. The mean ± SEM vales are presented and, where appropriate significance was determined using Student's *t* tests.

*Surface biotinylation*: Surface protein was isolated as previously described [8]. Briefly, HEK293 cells expressing SEP-mGluR7 or non-SUMOylatable mutant were labelled with NHS-Sulfo-SS-Biotin (0.15 mg/ml in PBS; Pierce) for 20 min at 4 °C, washed three times in PBS and neutralised with PBS containing 50 mM NH_4_Cl. After a final PBS wash, cells were lysed and solubilised. Fifty microliters of washed streptavidin agarose (Sigma) was incubated with 50–100 μg cleared lysate. Protein was eluted from the beads with 50–100 μl 2xSDS-PAGE loading buffer and Western blotted.

*Immunoprecipitation*: 200–400 μg of solubilised cleared neuronal lysate was incubated with 2–4 μg rabbit polyclonal mGluR7 antibody (Upstate) at 4 °C for 1–2 h. Fifty microlitres of Protein A beads were then added to each IP and allowed to incubate for another hour. After extensive washing with lysis buffer, protein was then eluted from the beads by addition of 50 μl 2xSDS-PAGE loading buffer and Western blotted.

*GFP-trap*: Washed GFP-trap A beads (Chromotek) were mixed with solubilised protein for 1 h at 4 °C before extensive washing and elution of the bound protein with 2xSDS-PAGE loading buffer and Western blotting.

*ERK assays*: HEK293 cells were transfected with 1 μg mGluR7 DNA and 24 h later complete DMEM (Dulbecco's modified Eagle medium (Gibco) containing 10% fetal calf serum, 2 mM l-glutamine and 100 U/ml penicillin/streptomycin) was removed and cells washed twice and incubated in unsupplemented DMEM to reduce basal serum-stimulated ERK activation for 12–16 h. DMEM was then replaced with either vehicle or mGluR agonist. After stimulation, DMEM was removed and cells lysed directly in 500 μl SDS-PAGE loading buffer. Lysates were sonicated briefly to reduce viscosity before analysis via Western blotting.

*Imaging*: Live cell imaging was performed as previously described [7]. 13 DIV cortical neurons were transfected with SEP-mGluR7 using lipofectamine 2000. Live cell imaging was performed 5 days after transfection using an inverted confocal microscope (Axiovert 200 M, Zeiss, Germany). Transfected (green) cells were selected and confocal sensitivity adjusted to allow both increases and decreases in fluorescence caused by pH changes to stay within the dynamic range. Fluorescence was excited at 488 nm and detected at 505 nm. Images were sampled every 30 s.

**Fig. 1:**
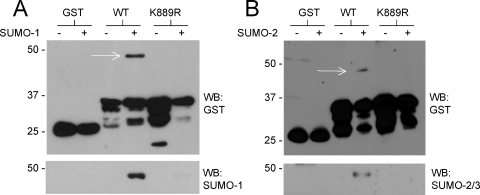
mGluR7 can be modified by both SUMO-1 and SUMO-2 at lysine 889 *in vitro*: The C-termini of mGluR7 wild-type or a mutant lacking the SUMOylation consensus lysine (K889R) were tested for SUMO modification in the bacterial SUMOylation assay with either SUMO-1 (A) or SUMO-2 (B). Crude bacterial lysates were either probed for GST (top panels) or GST-mGluR7 purified and probed for SUMO-1 or SUMO-2 (bottom panels). SUMO-modified mGluR7 is indicated by an arrow. K889 appears to be the only SUMOylatable lysine in the C-terminus of mGluR7, and this lysine can be modified by both SUMO-1 and SUMO-2.

mGluR7 contains a SUMOylation consensus motif in the intracellular C-termini that can be SUMOylated by SUMO-1 in bacterial SUMOylation assays [23]. Here we show that mGluR7 can also be SUMOylated *in vitro* by SUMO-2. To determine the site of SUMO modification we constructed a mutant of GST-ct-mGluR7 in which the positively charged consensus lysine residue (K889) was changed to a positively charged non-SUMOylatable arginine residue. As shown in Fig. 1, a single ∼50 kDa higher molecular weight species was observed with SUMO-1 (A) or SUMO-2 (B) in the wild-type GST-ct-mGluR7 but not the K889 mutant. The lower panel shows that this higher band was specifically recognised by anti-SUMO antibodies. Thus, mutation of the consensus lysine residue abolished modification by both SUMO-1 and SUMO-2.

**Fig. 2:**
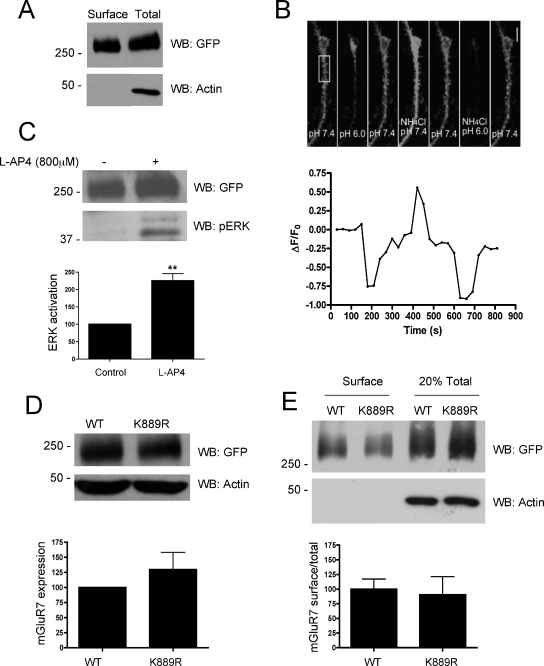
Expression and surface trafficking of a wild-type and non-SUMOylatable SEP-mGluR7 in HEK293 cells: (A) SEP-mGluR7 was transfected into HEK293 cells. Thirty-six hours post-transfection, surface proteins were labelled with biotin, the cells lysed and biotinylated surface proteins were isolated on streptavidin beads and subjected to Western blotting alongside total lysate for GFP (SEP). (B) SEP-mGluR7 was transfected into 13DIV primary cortical neurons and imaged for SEP expression 5 days later (upper panel). SEP fluorescence was reduced with a transient exposure to pH 6.0 buffer consistent with the majority of the signal arising from surface expressed SEP-mGluR7. Application of ammonium chloride, which collapses pH gradients across the plasma membrane and transiently equilibrates intracellular compartments to the extracellular pH increased fluorescence showing the total SEP signal (pH 7.4) or minimum signal (pH 6). The fluorescence of the region boxed in the upper panel is shown graphically in the lower panel. (C) SEP-mGluR7 is functional in HEK293 cells, as assayed for its ability to activate the ERK pathway. Data are the mean ± SEM, **p < 0.01 (Student's *t*-test, *n* = 3). (D) SEP-mGluR7 WT or K889R was transfected into HEK293 cells. Thirty-six hours post-transfection cells were lysed in SDS-PAGE loading buffer and subjected to Western blotting for GFP (mGluR7) or β-actin to ensure equal loading. (E) SEP-tagged mGluR7 WT or K889R was transfected into HEK293 cells. Thirty-six hours post-transfection, surface proteins were biotinylated and cells lysed. Equal amounts of protein were then incubated with streptavidin beads to isolate labelled surface proteins and subjected to Western blotting alongside total lysate for either GFP (mGluR7) or β-actin to confirm the specificity of surface labelling. The proportion of total mGluR7(K889R) at the surface is shown graphically normalized to the wild-type. The blots are representative of three separate experiments and the mean ± SEM are shown in the bar graphs; differences were not significant in Student's *t*-test.

To monitor the surface expression, lateral diffusion and endocytosis of mGluR7 we constructed full-length mGluR7(WT) and mGluR7(K889R) tagged with super-ecliptic pHluorin (SEP) at the extracellular N-terminal. SEP is a variant of GFP that is recognised by our anti-GFP antibody but which has been mutated to render the fluorescent signal sensitive to pH [1]. Consistent with a previous report [15], expression and surface trafficking of SEP-mGluR7 was confirmed in HEK293 cells by surface biotinylation and Western blotting (Fig. 2A), and via live cell imaging in transfected cortical neurons (Fig. 2B). Further, ERK assays in transfected HEK293 assays indicate that SEP-mGluR7 is functionally active (Fig. 2C).

**Fig. 3:**
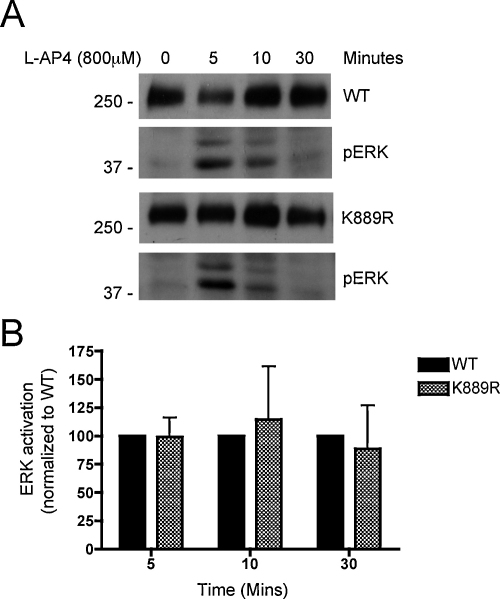
ERK activation downstream of a non-SUMOylatable mGluR7 is identical to wild-type mGluR7. (A) SEP-mGluR7 WT or K889R was transfected into HEK293 cells. Twenty-four hours post-transfection, cells were serum-starved for at least 6 h to reduce basal ERK signalling. Cells were then stimulated with the Group III mGluR agonist L-AP4 for the times indicated, lysed in SDS-PAGE loading buffer and subjected to Western blotting for GFP (mGluR7) or phospho-ERK. (B) ERK activation at the times indicated is shown normalized to the wild-type. Data are shown as mean ± SEM for three separate experiments. There is no significant difference in the duration or extent of ERK activation between WT and non-SUMOylatable mGluR7.

To determine whether expression levels of mGluR7 were affected by mutation of the SUMOylatable lysine (K889), HEK293 cells were transfected with the WT or non-SUMOylatable mGluR7(K889R) mutant and total lysates were Western blotted for mGluR7 (Fig. 3A). Total levels of mGluR7 were unaffected by the K889R mutation. To assess surface expression we used membrane impermeant Sulfo-NHS-Biotin, streptavidin pulldowns and subsequent Western blotting of surface versus total fractions. For quantification, the level of surface expressed mGluR7 was normalized to the total mGluR7 for each experimental condition. The bar graph (Fig. 3B) shows the proportion of surface expressed mGluR7(K889R) normalized to the proportion observed for wild-type mGluR7. Similar levels of surface expression were observed for both the mGluR7(WT) and the SUMOylation-deficient mutant mGluR7(K889R).

**Fig. 4:**
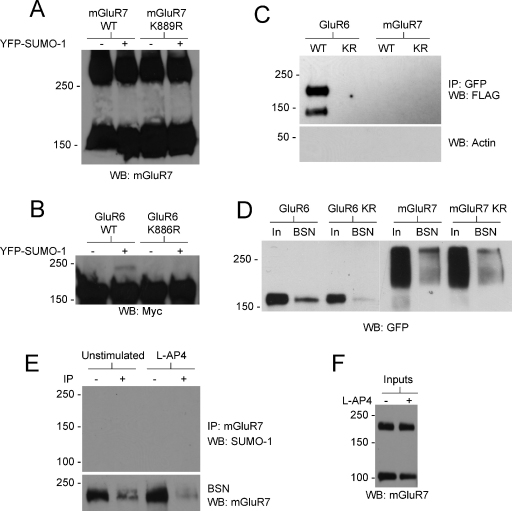
SUMOylation of mGluR7 was not detected in HEK293 cells or neurons. (A) SEP-mGluR7 was co-transfected into HEK293 cells in the presence or absence of FLAG-Ubc9 and YFP-SUMO-1. Cells were lysed in SDS-PAGE loading buffer and subjected to Western blotting for mGluR7. The two bands represent monomers and dimers of mGluR7, but a band representing a SUMO-modified form of mGluR7 was not detected. (B) As (A), except cells were transfected with a myc-tagged form of the kainate receptor subunit GluR6 or the non-SUMOylatable K886R mutant. Note the presence of a SUMOylated higher molecular weight form of GluR6 upon expression of YFP-SUMO-1 which is not present for the K886R mutant. (C) HEK293 cells transfected with YFP-GluR6, SEP-mGluR7 or their non-SUMOylatable mutants (denoted as KR) along with FLAG-Ubc9 and FLAG-SUMO-1. GFP-tagged proteins were immunoprecipitated with GFP-trap beads and Western blotted for FLAG. (D) Inputs and equivalent amounts of bead supernatant (BSN) from IPs shown in (C) showing successful depletion (pulldown) of target proteins. (E) 14DIV cortical neurons were lysed, mGluR7 immunoprecipitated and blotted for SUMO-1 (upper panel). Bead supernatant (BSN) was also run alongside an equivalent amount of input to determine pulldown of mGluR7 was successful. (F) Inputs corresponding to the IPs shown in (E).

Group III mGluRs signal in part via activation of the ERK MAP kinase pathway [6]. To determine whether the extent and duration of ERK activation differed between a wild-type and non-SUMOylatable mGluR7(K889R), HEK293 cells were transfected with either SEP-mGluR7(WT) or SEP-mGluR7(K889R), stimulated with the group III mGluR-specific agonist L-AP4 (800 μM) for various times and lysates blotted for phospho-ERK. Both the magnitude of the initial response and the length of the response (desensitization) were indistinguishable between a wild-type and non-SUMOylatable mGluR7 (Fig. 4).

The lack of identifiable affect in preventing mGluR7 SUMOylation led us to query whether mGluR7 is indeed a *bona fide* substrate for SUMOylation. To address this directly HEK293 cells were transfected with plasmids encoding SEP-mGluR7(WT) or SEP-mGluR7(K889R) together with FLAG-Ubc9 and YFP-SUMO-1 and total cell lysates were analysed by Western blotting. We did not detect any SUMOylation of mGluR7, despite clear SUMOylation of the previously reported SUMO substrate GluR6 in the same assay [10] (Fig. 4A,and B). To increase the sensitivity of the assay, cells were lysed and SEP-mGluR7 immunoprecipitated on GFP-trap beads and blotted for FLAG-SUMO. Again, although robust SUMOylation of GluR6 was detected no SUMOylation of mGluR7 was observed (Fig. 4C and D). One possible explanation might be that HEK293 cells are not an appropriate system to analyse SUMOylation of mGluR7, for example because of insufficient levels of an E3. Therefore, we performed immunoprecipitation experiments from cultured cortical neurons. 14 DIV cortical cultures were lysed, mGluR7 immunoprecipitated and Western blotted for SUMO-1. As shown in Fig. 4E and F, mGluR7 was present in the cell lysates and was depleted in the bead-supernatant indicating immunoprecipitation of mGluR7 under these conditions, but no SUMOylated mGluR7 was detected.

Previous results, including from our own lab, have shown that group III mGluRs can bind components of the SUMOylation machinery and that in recombinant systems the soluble C-terminal domains are robustly SUMOylated [20,23]. These data suggested that SUMOylation of group III mGluRs might play roles in regulating presynaptic release and in mGluR-dependent forms of metaplasticity. Here we show that lysine 889 in mGluR7 is SUMOylated by SUMO-1 and SUMO-2 *in vitro* and we engineered a SUMO deficient mGluR7(K889R) mutant to define the roles of SUMOylation in the full-length receptor. Expression of wild-type or non-SUMOylatable mGluR7 in HEK293 cells, which lack endogenous mGluR7, allowed direct comparison between the properties of the recombinant receptors. Surprisingly, mGluR7(WT) and mGluR7(K889R) were indistinguishable in total receptor expression, surface trafficking and ERK activation making it unlikely that this modification regulates receptor stability, mGluR7 trafficking or signalling.

Another study has reported that mGluR7 is capable of interacting with Ubc9 in a yeast two-hybrid screen [19]. Yeast two-hybrid assays are undoubtedly a useful tool for the identification of potential protein interactions but they can fail to detect known interactions and often identify potential interacting partners that do not genuinely interact *in vivo* due to their structure, regulation or cellular localisation in mammalian cells [13]. Further, while interaction with Ubc9 is generally a good indication of potential SUMO modification, it is unclear whether this is necessarily the case for membrane proteins. Nuclear proteins that interact with Ubc9 have a high probability of being targets for SUMOylation due to the high levels of SUMO and Ubc9 in the nucleus. Indeed, it has been reported that a SUMOylation consensus motif and a nuclear localisation signal are all that is required for SUMOylation of a substrate protein to occur [17]. Levels of Ubc9 and SUMO at the cell periphery are likely to be lower, so in the case of plasma membrane proteins, identifying an *in vitro* interaction with Ubc9 may not necessarily mean that the protein of interest is a genuine SUMO substrate. For similar reasons our identification of robust SUMOylation of each of the group III mGlu receptors in the bacterial SUMOylation assay [23] must also be treated with caution.

Although extensively used, HEK293 cells might not accurately represent the situation in neurons. For example, it is possible that neuron-specific factors could regulate the stability, surface trafficking or G protein-coupling and that interactions with these factors are required to define a functional consequence of mGluR7 SUMOylation. This is exemplified by our observation mGluR7 does not undergo agonist-induced endocytosis in HEK293 cells (data not shown). Similarly, it has been reported that mGluR7 does not undergo agonist-induced endocytosis in HeLa cells [9]. Thus, the rapid and robust activity-dependent endocytosis of mGluR7 reported in neurons could be mediated by a neuron-specific mechanism. In addition, it is well established that mGluRs are relatively insoluble, they have a tendency to run as dimers or higher order oligomers and they are difficult to immunoprecipitate and immunoblot [3]. Despite the fact that in *in vitro* assays we observe robust SUMOylation, we have been unable to verify mGluR7 SUMOylation in either heterologous cells or neurons although in the same experiments we did observe SUMOylation of GluR6 as previously reported [10]. Therefore, while we cannot definitively rule out the possibility that mGluR7 is SUMOylated, our results appear to question whether mGluR7 is a physiologically relevant SUMO substrate.

Several papers have discussed the potential role of SUMOylation in group III mGluR function. For example, it has been proposed that SUMOylation of mGluR7 may explain how PICK1 can still be partially immunoprecipitated with mGluR7 from transgenic mice lacking the mGluR7 PDZ ligand [24]. Further, reviews of SUMOylation have cited group III mGluRs as examples of surface-expressed SUMO substrates (for example [4,11,18,22]). While our data support previous observations that the C-terminus of mGluR7 is SUMOylated, our lack of confirmatory results using the full-length receptor suggest these reports may be premature and, in the worst case scenario, imply that group III mGluR SUMOylation may be restricted to recombinant systems and not occur at native receptors under physiologically relevant conditions. Thus, further work will be required to confirm the SUMOylation status of the group III mGluRs and define the roles, if any, SUMOylation plays on these receptors.
